# Retracted: Marathon Runner with Acute Hyponatremia: A Neurological Disorder

**DOI:** 10.1155/2019/3954675

**Published:** 2019-03-26

**Authors:** Case Reports in Emergency Medicine

At the request of the authors, the article titled “Marathon Runner with Acute Hyponatremia: A Neurological Disorder” [1] has been retracted. The article was previously published as “Kormann R, Philippart F, Hubert S, Bruel C, “Marathon Runner with Acute Hyponatremia: A Neurological Disorder”. Emerg Med (Los Angel) 2:106, (2012). doi:10.4172/2165-7548.1000106” [2].
